# Static Stretching Alters Neuromuscular Function and Pacing Strategy, but Not Performance during a 3-Km Running Time-Trial

**DOI:** 10.1371/journal.pone.0099238

**Published:** 2014-06-06

**Authors:** Mayara V. Damasceno, Marcos Duarte, Leonardo A. Pasqua, Adriano E. Lima-Silva, Brian R. MacIntosh, Rômulo Bertuzzi

**Affiliations:** 1 Endurance Performance Research Group, School of Physical Education and Sport, University of São Paulo, São Paulo, São Paulo, Brazil; 2 Biomedical Engineering, Federal University of ABC, Santo André, São Paulo, Brazil; 3 Sports Science Research Group, Department of Physical Education and Sports Science, Federal University of Pernambuco, Vitoria de Santo Antão, Pernambuco, Brazil; 4 Human Performance Laboratory, Faculty of Kinesiology, University of Calgary, Calgary, Alberta, Canada; The University of Queensland, Australia

## Abstract

**Purpose:**

Previous studies report that static stretching (SS) impairs running economy. Assuming that pacing strategy relies on rate of energy use, this study aimed to determine whether SS would modify pacing strategy and performance in a 3-km running time-trial.

**Methods:**

Eleven recreational distance runners performed *a*) a constant-speed running test without previous SS and a maximal incremental treadmill test; *b*) an anthropometric assessment and a constant-speed running test with previous SS; *c*) a 3-km time-trial familiarization on an outdoor 400-m track; *d* and *e*) two 3-km time-trials, one with SS (experimental situation) and another without (control situation) previous static stretching. The order of the sessions *d* and *e* were randomized in a counterbalanced fashion. Sit-and-reach and drop jump tests were performed before the 3-km running time-trial in the control situation and before and after stretching exercises in the SS. Running economy, stride parameters, and electromyographic activity (EMG) of vastus medialis (VM), biceps femoris (BF) and gastrocnemius medialis (GA) were measured during the constant-speed tests.

**Results:**

The overall running time did not change with condition (SS 11:35±00:31 s; control 11:28±00:41 s, p = 0.304), but the first 100 m was completed at a significantly lower velocity after SS. Surprisingly, SS did not modify the running economy, but the iEMG for the BF (+22.6%, p = 0.031), stride duration (+2.1%, p = 0.053) and range of motion (+11.1%, p = 0.0001) were significantly modified. Drop jump height decreased following SS (−9.2%, p = 0.001).

**Conclusion:**

Static stretch impaired neuromuscular function, resulting in a slow start during a 3-km running time-trial, thus demonstrating the fundamental role of the neuromuscular system in the self-selected speed during the initial phase of the race.

## Introduction

The manner in which runners distribute their speed during a competition is defined as pacing strategy [1]. It has been widely recognized that the pacing strategy adopted by athletes can substantially impact performance in long-distance running [1]. During these competitive events, endurance athletes usually adopt a pacing strategy with a speed distribution consisting of three distinct phases. These phases are characterized by a fast start, followed by a period of slower speed during the middle of the race, and a significant increase in running speed towards the end [2], [3]. It has been previously demonstrated that the strategy with the highest speeds reached during start phase (i.e. fast start) is advantageous for increasing oxygen uptake early and decreasing the use of anaerobic energy reserves [4]. Additionally, a fast acceleration relies on the ability to generate high forces, suggesting an importance of neuromuscular system for the start phase [5].

The rating of perceived exertion (RPE) [6] can be evaluated during a running race in order to verify how effort and perceived difficulty relate to actual speed. More recently, Faulkner et al. [7] observed that when speed distribution during long-distance running was characterized by the triphasic speed distribution profile described above (so-called “*U* shaped” pacing strategy), the RPE increases linearly. It has been hypothesized that this linear profile of RPE during a time trial test reflects a centrally-regulated control system that is dependent on a patterned disturbance to muscular homeostasis. It is believed that this system regulates the pattern and magnitude of muscular activation to maintain the physiological strain at a tolerable level and to prevent premature exercise termination [8].

In addition to a centrally-regulated system, it has been proposed that pacing strategy can be influenced by some physiological feedback [9], [10] and neuromuscular [11] performance changes. For example, Lima-Silva et al. [12] observed that long-distance runners who had higher running economy (RE) were able to adopt a more aggressive pacing strategy, employing faster velocities at the start phase (first 400 m) in a 10-km running race. In addition, it has been proposed that neuromuscular factors related to force production and muscular recruitment must also be considered when investigating the determinants of endurance performance [2], [13]. Considering the highest speeds reached during the start phase, it seems plausible to assume that the neuromuscular variables related to force production might also be important determinants of pacing strategy and success in long-distance events.

Static stretching (SS) is commonly used as part of a warm-up routine for athletes, yet SS has received considerable attention because it seems to have an acute negative effect on activities that are strength- and power-dependent [14], [15]. Previous studies have reported that an acute session of SS resulted in impairments on sprint performance [16], [17] and in jump height [14], [15], [18]. For example, Sayers et al. [17] observed a negative effect on the acceleration phase of a sprint test after SS. This impaired performance induced by SS treatment may be related to an inability to maximally activate muscle. Avela et al. [19] reported that there were significant decreases in maximal voluntary contraction (23.2%) and EMG (19.9%) following 1 h of passive stretching of the triceps surae. Collectively, these data suggest that SS results in reduced capacity of the skeletal muscle to produce explosive force and this could result in a reduction in speed during the acceleration phase of a long-distance race. Furthermore, SS has been reported to increase the energy cost of running [20], but there is not universal agreement on this [21]. Collectively, these consequences of SS would be expected to alter pacing strategy and consequently the performance during a 3 km time-trial.

To date, no study has considered the potential negative influence of SS on pacing strategy during a long-distance running event. Since force generating capability is an important determinant of endurance performance [13], it is attractive to suspect that prior SS treatment could alter the acceleration phase during a long-distance event. Thus, the main objective of the current investigation was to analyze the acute effect of SS on pacing strategy adopted during a 3-km running time-trial. It was hypothesized that SS would increase the energy cost of running, reduce the capacity of lower limbs to produce explosive force, and reduce the initial speed in a 3-km running time-trial.

## Methods

### Participants

Eleven male, recreationally trained long-distance runners (mean age: 35.7±6.1 years; height: 1.76±0.08 m; mass: 79.7±11.3 kg; maximal oxygen uptake: 51.0±3.0 ml•kg^−1^•min^−1^) volunteered to participate in this study. All participants regularly competed in 10-km running races at regional levels, and their best performances in 10-km competitions ranged from 35–45 minutes. They were included if they had been training for the last 2 years without interruption and for at least three times per week with a minimum weekly volume above 30 km. The study was conducted at the beginning of the year during a non-competitive period. The subjects performed only low-intensity continuous aerobic training (∼60% maximal oxygen uptake) and reported no previous strength or plyometric training experience. None of the participants were receiving any pharmacological treatments or had any type of neuromuscular disorder or cardiovascular, respiratory or circulatory dysfunction. The participants received a verbal explanation about the possible benefits, risks and discomfort associated with the study and signed a written informed consent before participating in the study. Procedures were in accordance with the Helsinki Declaration of 1975, and this investigation was approved by the Ethics and Research Committee of the University of São Paulo.

### Experimental design

**Figure 1:**
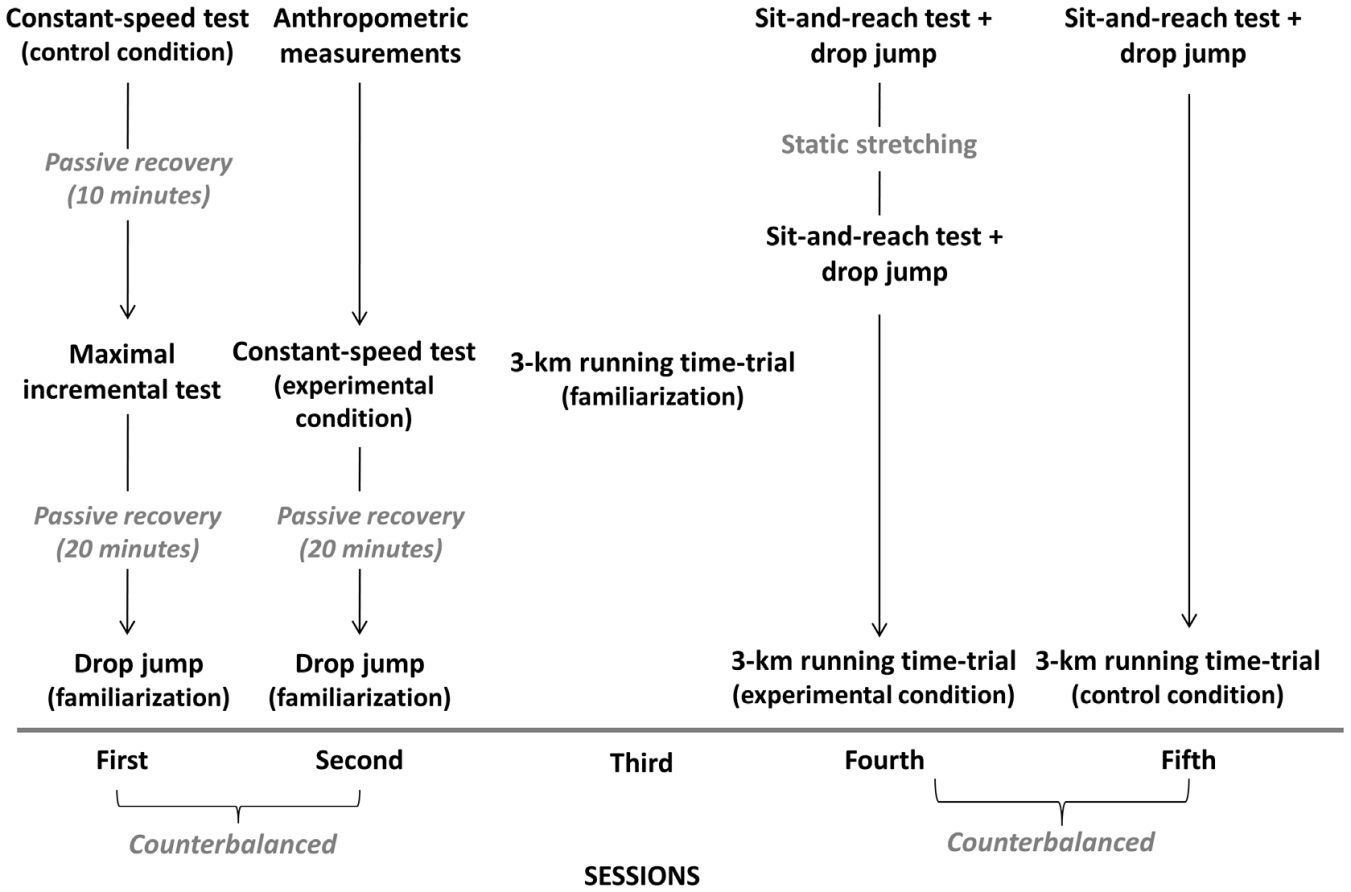
Pictorial view of the experimental design.

Participants visited the laboratory on five separate occasions, with at least 48 h between sessions, over a three-week period. Figure 1 gives a pictorial view of the experimental design. In the first session, the participants completed one 6-min, constant-speed test by running at 12 km•h^−1^ without previous SS treatment (control condition) and a maximal incremental treadmill test. Ten minutes of passive recovery was allowed between these two tests. In the second session, anthropometric measurements and one 6-min, constant-speed running test at 12 km•h^−1^ with previous SS treatment (experimental condition) was performed. Drop jump familiarizations were conducted at the end of the first and second visits after 20 minutes of passive recovery. The order of presentation for components of the first and second visits was counterbalanced. In the third session, the participants performed a 3-km time trial test familiarization on an outdoor 400-m track. In the fourth and fifth sessions, the participants performed a 3-km time trial test either with or without previous SS treatment. For the experimental condition, the runners performed a sit-and-reach test and a drop jump before and after SS to determine the impact of SS on range of motion and the stretch-shortening cycle, respectively. These tests were also performed in the control condition before the 3-km time trial. The order of presentation for components of the fourth and fifth visits was counterbalanced. The duration of each experimental session was approximately 50 minutes. All of the tests were performed at the same time of day for a given subject, at least 2 h after the most recent meal. The subjects were instructed to maintain their training program during the study period, but they were asked to refrain from any exhaustive or unaccustomed exercise during the preceding 48 h of any experimental test and to refrain from taking nutritional supplements during the experimental period.

### Laboratory tests

#### Anthropometric measurements

Anthropometric measurements were performed according to the procedures described by Lohman [22]. Participants were weighed to the nearest 0.1 kg using an electronic scale (Filizola, model ID 1500, São Paulo, Brazil). Height was measured to the nearest 0.1 cm using a stadiometer. Skinfold thickness was measured to the nearest 0.2 mm at eight body sites (i.e., triceps brachii, suprailiac, abdominal, chest, subscapular, midaxillar, anterior thigh and calf) using a Harpenden caliper (West Sussex, United Kingdom). The median of three values was used for data analysis. Measurements were performed by an experienced investigator. Body density was estimated using the equation of Jackson and Pollock [23], and body fat was estimated using the equation of Brozek et al. [24].

#### Maximal incremental test

A maximal incremental running test was performed on a motorized treadmill (model TK35, CEFISE, Nova Odessa, Brazil). After a warm-up at 8 km•h^−1^ for 5 min, the speed was increased by 1 km•h^−1^ every minute until exhaustion. The participants received strong verbal encouragement to continue as long as possible. Expired gases were measured by a metabolic measurement cart (Cortex Metalyzer 3B, Cortex Biophysik, Leipzig Germany) to determine VO_2_ and carbon dioxide output (VCO_2_) and were subsequently averaged over 30-s intervals throughout the test. Before each test, the metabolic cart was calibrated using ambient air and a gas containing 12% O_2_ and 5% CO_2_. The turbine flowmeter was calibrated using a 3-L syringe (Quinton Instruments, Seattle, WA, USA). Heart rate (HR) was monitored during the test with a HR transmitter (model S810, Polar Electro Oy, Kempele, Finland) coupled to the gas analyzer. Maximal heart rate (HRmax) was defined as the highest value obtained at the end of the test. Maximal oxygen uptake (VO_2_max) was determined when two or more of the following criteria were met: an increase in VO_2_ of less than 2.1 ml•kg^−1^•min^−1^ between consecutive stages, a respiratory exchange ratio greater than 1.1, and reaching ±10 bpm of the maximal age-predicted heart rate [25].

#### Constant-speed test

To analyze the impact of an SS bout on running parameters, the participants performed two constant-speed tests (experimental *vs*. control condition) on a motorized treadmill (model TK35, CEFISE, Nova Odessa, Brazil). Before the tests, the athletes performed a standardized warm-up consisting of a 5-min run at 8 km•h^−1^, followed by a 3-min passive recovery. The treadmill speed was adjusted to 12 km•h^−1^ after the warm-up, and the subjects ran for 6 minutes at this speed. The test began with the participant's feet astride the moving belt and hands holding the handrail. For the experimental condition, the athletes performed the test immediately after the SS treatment. The VO_2_ over the final 30 seconds was taken as the steady-state VO_2_ for that speed. Running economy (RE) was defined as described by Fletcher et al. [26]. Taking the average RER over the same 30 seconds, the caloric equivalent of the VO_2_ (kcal•l^−1^O_2_) was determined [27], and the caloric unit cost was calculated using equation 1:
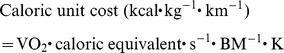
(Eq.1)


Where VO_2_ is measured in liters per minute, caloric equivalent is in kilocalories per liter, speed *(s)* is in meters per minute, body mass (BM) is in kilograms, and K is 1000 m•km^−1^.

The resting metabolic rate was not subtracted because it cannot be confirmed that resting metabolic demand continues at the same rate while running. Stride parameters and EMG signals were simultaneously measured from the left leg during the last 10 s of the constant-speed tests. Disposable dual Ag/AgCl snap electrodes with a 1 cm diameter and a 2-cm center-to-center spacing (Noraxon, Scottsdale, AZ, USA) were placed on the belly of the vastus medialis (VM), biceps femoris (BF) and gastrocnemius medialis (GA) before starting the tests. The guidelines published by SENIAM [Surface Electromyography for the Non-Invasive Assessment of Muscles (SENIAM)] were followed for skin preparation, electrode placement and orientation. Electrode positions were marked with small ink tattoos on the skin during the first testing session to ensure that electrode placement over the entire experimental period would be consistent [28]. The EMG signals were recorded with a telemetric EMG system, which had a gain of 1000 times, a bandwidth (−3 dB) over 10 to 500 Hz, and a common mode rejection ratio >85 dB and was relayed to the computer via a 16-bit A/D converter (Telemyo 900, Noraxon, Scottsdale, AZ, USA).

The EMG data were band-pass filtered at 20–400 Hz, and an envelope representing the muscle activation was determined using a moving RMS filter with a window of 50 ms. The period of activation of each muscle during a stride was determined as the period where the signal was above a threshold of 15% of the maximum activity of that muscle during the trial for at least 100 ms. These parameters were selected based on the signal-noise relationship of the EMG data and were visually verified to correctly identify periods of muscle activation. For each bout of EMG activation, we calculated the integrated EMG (iEMG), defined as the area under the EMG versus time curve divided by the period of activation.

A video camera (GR-DVL9800U, JVC Inc., Wayne, NJ, USA) was used to record frontal plane images at 120 Hz during the last 10 s of running. Stride parameters were measured simultaneously with EMG using Noraxon's Myoresearch software (Version 1.08). Using frame-to-frame video analysis, ten steps were analyzed. Contact time was defined as the time from ground contact of the left foot until the time that the same foot left the ground. The flight time was determined from the time when the left foot left the ground to the time prior to the next contact of the same foot. The stride time was defined as the time from ground contact of one foot to the next ground contact of the same foot (i.e., contact time+ flight time). Given that contact time was ∼250 ms and images were captured at 120 Hz sampling rate, the experimental uncertainty of digital instruments in our case was ∼8 ms, corresponding to an uncertainty of 3.2%. This is acceptable considering the between subjects variability of the measurements was larger.

### Field tests

#### 3-km running test

To analyze the impact of SS on pacing strategy, participants individually performed a 3-km run on an outdoor 400-m track on three different days (familiarization, experimental, and control conditions). These running time-trial tests were performed with an interval of at least 48 h between them. Before each time-trial, the participants did 10 minutes of warm-up at 8 km•h^−1^. They were instructed to maintain regular water consumption within six hours of testing, and water was provided *ad libitum* during the entire event. The participants were instructed to finish the race as quickly as possible, as they would in a competitive event. Verbal encouragement was provided during the entire event. However, runners were not advised of their lap splits. Speed was registered every 100 m via a global positioning system (GPS Forerunner 305, Garmin, Kansas City, Oregon, EUA). RPE was reported by participants every 400 m using the Borg 15-point scale [6]. Copies of this scale were laminated and reduced to 10 cm by 5 cm, and they were affixed to the wrist of the dominant arm of the individuals. The 3-km running tests were performed at the same time of day and in similar conditions. Ambient temperature and humidity were provided by the Institute of Astronomy, Geophysics and Atmospheric Sciences of the University of São Paulo, Brazil. The mean ± SD values for temperature and humidity were 24.1±3.9°C and 63.0±9.7%, respectively.

The sit-and-reach and drop jump tests were performed before and immediately after the SS in the 3-km running time-trial session with previous SS, and they were performed before the 3-km running time-trial session without SS. In the former situation, these tests were performed to verify the effectiveness of SS on the range of motion and capacity of lower limbs to produce explosive force, respectively. The sit-and-reach test was used because it provides a global measure of hamstring, hip, and lower back flexibility [29]. The participants sat with their bare feet pressed against the sit-and-reach box. Knees were extended, and the right hand was positioned over the left. Participants were then asked to push a ruler transversely located over the box as far as possible on the fourth bobbing movement. Each subject performed 3 trials of the sit-and-reach test, and the best trial was used for analysis. After the sit-and-reach test, the participants were instructed to perform a drop jump. The jump height was determined by flight time, which was measured by a contact mat (MultiSprint, Hidrofit, Brazil). Athletes stepped off a 40 cm box and attempted to achieve the greatest vertical height with a short ground contact time (close to 200 ms) [30]. Subjects were instructed to minimize knee flexion and extension during the drop jump, and a demonstration was provided by the investigators. All jumps were performed with hands on hips, and five repetitions were performed with a 30-s rest between jumps. The largest and smallest values were rejected, and the average of the remaining 3 jumps was calculated and used for statistical analysis.

### Intervention protocol

#### Static stretching

The stretching treatment used in the present study was similar to that described in Samogin-Lopes et al. [31]. The SS involved seven different exercises for the lower limbs, including 5 unassisted and 2 assisted exercises. Briefly, the exercises performed were unassisted straight-leg stand and toe touch, unassisted standing quadriceps stretching, unassisted hamstrings and back stretching, unassisted hurdler's stretching, unassisted standing calf stretching, assisted quadriceps and hip stretching, and assisted thigh stretching. Each exercise was performed three times, and each time the stretching position was maintained for 30 seconds. The magnitude of stretch was sufficient to yield a score of 8–9 on the Borg CR10 scale [6]. The total duration of time required for completion of the SS treatment was approximately 20 minutes.

#### Statistical analyses

Data normality was assessed by the Shapiro-Wilk test, and all variables showed a normal distribution. All data are reported as means and standard deviations (±SD). A paired *t-* test was used to determine differences between non-stretching and SS treatments for RE, EMG, drop jump height, sit-and-reach test, flight time, contact time and stride time. Repeated measures analysis of variance with two factors (distance x condition), followed by a Bonferroni adjustment to compare the alterations in the speed and RPE during the 3-km time trials. The level of significance was set at p≤0.05. All statistical analyses were conducted using the SPSS statistical package (version 16.0, Chicago, USA). Smallest worthwhile change (SWC; clinically beneficial effect) was also determined for performance parameters using the method described by Batterham and Hopkins (32). A Cohen's unit of 0.2 was used to determine the SWC. The uncertainty in the effect was expressed as difference and 90% confidence limits (difference ±95% CL) and as likelihoods that the true value of the effect represents substantial change (harm or benefit). When clear interpretation could be made, a qualitative descriptor was assigned to the following quantitative chances of benefit: <1%, almost certainly not; 1–5%, very unlikely; 5–25%, unlikely or probably not; 25–75%, possibly or may be; 75–95%, likely or probably; 95–99%, very likely; >99%, almost certainly (32). Where the chances of benefit or harm were both calculated to be ≥5%, the true effect was deemed unclear.

## Results

### Laboratory tests

Table 1 shows the anthropometric and physiological characteristics of the participants. Table 2 shows the variables measured during the RE tests. No significant differences were observed in the caloric unit cost of running (p = 0.128) or HR between the conditions (p = 0.317). The iEMG for the BF muscle was significantly higher in the SS condition, compared to the control condition (p = 0.031). No significant changes in iEMG were observed in either VM (p = 0.419) or GA (p = 0.212). The stride time was significantly longer in the SS condition (p = 0.053) than in the control, but no differences were observed for contact time or flight time.

**Table 1:** Anthropometric and physiological characteristics of the participants.

Variable	Mean ± SD
Age (years)	35.7±6.1
Height (cm)	173.3±9.0
Body mass (kg)	67.9±7.4
Body fat (%)	10.0±2.7
VO_2_max (ml.kg^−1^.min^−1^)	51.0±3.0
HRmax (beats.min^−1^)	184±6
RERmax	1.2±0.1

HRmax: maximal heart rate, RERmax: maximal respiratory exchange ratio.

**Table 2:** Variables measured during the constant-speed tests with or without previous static stretching.

	Control	Static stretching
RE (ml.kg^−1^.min^−1^)	41.3±2.8	40.4±3.0
CUC (kcal.kg^−1^.km^−1^)	1.03±0.07	1.00±0.08
iEMG_VM_(µV)	60±21	64±23
iEMG_GA_ (µV)	77±27	95.3±38
iEMG_BF_(µV)	73±26	94±31*
Contact time (ms)	256±26	250±32
Flight time (ms)	443±42	452±37
Stride time (ms)	697±41	710±41*

Values are means ± SD; RE: running economy; CUC: caloric unit cost; VM: vastus medialis; GA: gastrocnemius medialis; BF: biceps femoris. *Significantly different from control condition (p≤0.05).

### Field tests

**Figure 2:**
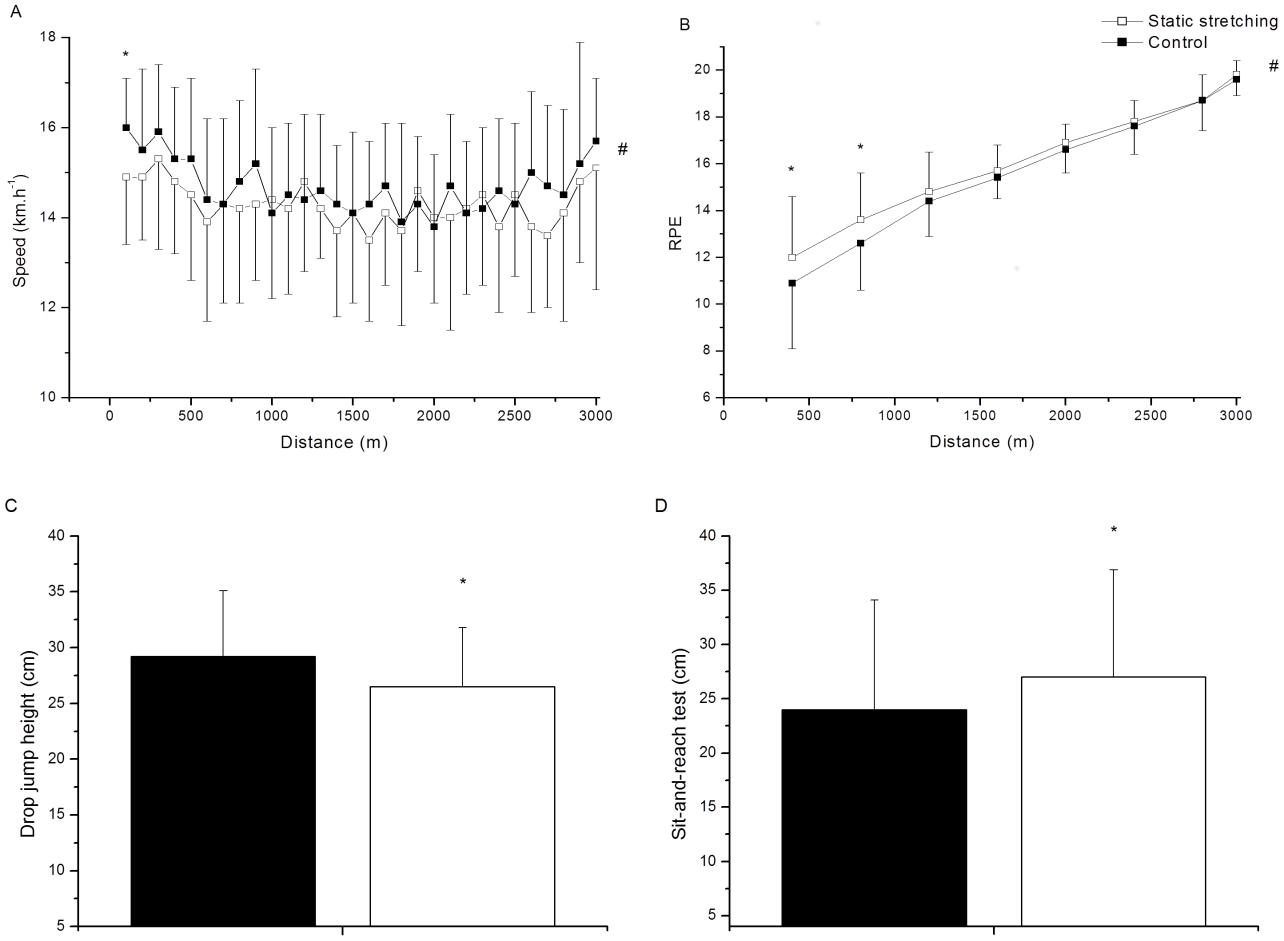
Variables measured with and without previous static stretching treatment. A and B panels show the running speed and rating of perceived exertion during a 3-km running time-trial, respectively. C and D panels show the drop jump and sit-and-reach tests performed prior to and immediately after static stretching treatment. * Significantly different from control situation (p≤0.05). #Significant difference over time in each condition (p≤0.05).

Variables measured during time-trial tests performed with and without previous static stretching are presented in figure 2. The speed-distance curve during 3-km running showed a classical *U*-shape in both conditions. It was detected that the first section (100 m) was completed at a significantly slower speed in the SS condition (very likely harmful, −1.1±1.0 km.h^−1^ 95% CL), compared with the control condition (p = 0.036). However, the overall running time to cover 3-km running during the control condition (11:28±00:41min:s) was not significantly different from that during the SS condition (11:35±00:31min:s) (trivial, 7.0±13.9 s 95% CL). The RPE increased significantly over time in both conditions (p = 0.001). The RPE in the SS condition was statistically greater than that in the control condition only during the first 800 m (p = 0.019). Following SS, the athletes also demonstrated reduced drop jump height (p = 0.001) and improved performance on the sit-and-reach test (p = 0.0001) relative to measures obtained prior to SS protocol. There were no differences in drop jump height (p = 0.351) and sit-and-reach test (p = 0.262) before the 3-km running when the control and experimental situations were compared.

## Discussion

The main objective of the present study was to investigate the impact of SS on pacing strategy and performance during a 3-km running time-trial. To the best of our knowledge, this is the first study to analyze the influence of this exercise-induced impairment in neuromuscular function on the pacing strategy adopted during a long-distance run. The main finding was that the SS resulted in a slow-start strategy during a 3-km running time-trial. We also observed an impaired drop jump performance and a higher perceived exertion during the first 800 m.

Acute effects in neuromuscular function after SS treatments have led to changes in isometric peak torque, range of motion, height in vertical and drop jumps [14], [18], [33]. In the current study, it was found that an SS bout resulted in an 11% increase in the sit-and-reach test and a 9.2% decrease in drop jump height before the 3-km running time-trial. These findings are similar to previous investigations. Young and Elliott [34] demonstrated that SS significantly decreased drop jump performance by 6.9% in recreational athletes. Furthermore, Behm and Kibele [18] demonstrated that SS for lower limbs significantly decreased the drop jump height (−4.6%) and increased the range of motion measured by the sit-and-reach test (+12.1%) in physically active subjects. These data show that the magnitude of modification in flexibility and jump performance after SS treatment demonstrated in the current study was similar to that reported in the literature.

Data from the current study also showed that reduced ability to produce force after the SS protocol was accompanied by a higher iEMG of the BF and stride time during constant-speed running test. It is well recognized that BF activation plays a key role in the control of knee extension and in the generation of the knee flexion force in the late swing phase before foot contact during running [35]. Thus, the higher iEMG of the BF after the SS protocol might have reflected an increased motor unit recruitment in order to maintain the running mechanics and compensate the reduced passive forces that would otherwise have served this purpose. In turn, this increased muscle activation may have contributed to the perception of effort as indicated by the RPE.

Previous findings have indicated an inverse relationship between RE and flexibility [20]. In the present study, it was found that SS produced a significant increase in range of motion, as measured by the sit-and-reach test (3±2 cm), but the RE measured at 12 km•h^−1^ was not statistically altered. These data were similar to the study of Allison et al. [21], who showed that the statistical changes detected in range of motion (2.7±0.6 cm), isometric strength (−5.6±3.4%) and countermovement jump height (−5.5±3.4%) were not accompanied by changes in the RE after SS. Similarly, Hayes and Walker [36] observed that static and dynamic stretching improved range of motion, but both treatments had no impact on the RE. Therefore, it seems that acute improvement in range of motion after SS is not associated with modification in the RE.

In relation to pacing strategy, our results revealed that runners adopted a slow start after SS. This reduced running speed during initial phase of the 3-km running time trial seems to be related to lower ability to produce force, as evidenced by decreased drop jump height and increased stride time found after the SS protocol. Previous studies have suggested that the ability to produce force is an essential determinant of endurance performance without being necessarily related to energy demand of running (e.g. running economy) [37], [38]. This occurs because middle and long-distance runners must be able to maintain a relatively high speed over the course of a race [39]. In particular, the acceleration phase requires a great level of muscle contraction in order to overcome inertia. Because the SS have a negative acute effect on the neuromuscular system [14], [15], its deleterious effect might be more pronounced in the start phase of a long-distance running. These results are in accordance with previous studies that reported reduced running sprint performance after a SS protocol [16], [17]. Taken together, these findings suggest that the SS induces a slow start in a 3-km running time trial due its negative influence in the neuromuscular system, impairing the acceleration phase of a long-distance event.

Interestingly, our results showed that the slow start induced by SS treatment was accompanied by an increase in the RPE. It has been suggested that RPE may reflect increased motor unit recruitment [40]. It is believed that collateral innervations are sent directly from the motor to the sensory areas in the brain, contributing to the increase of the RPE response during exercise [41]. Thus, it is plausible to suggest that the brain might have interpreted the efferent signals from increased motor unit recruitment of the BF as the first cues for running speed adjustments. Based on this finding, it is plausible to suggest that the greater RPE found during the start phase after SS may reflect an increased neural drive resulting from intention to produce the same amount of force and thus maintain a high initial running speed. This is in agreement with a previous suggestion that RPE has a relevant role in the speed control during the start phase of a running race [42].

Results of the present study revealed that despite the slow start, runners were able to maintain the overall running performance. This is consistent with the idea that the effects of SS were overcome during the event. Ryan et al. [43] showed that two 30-second bouts of SS were sufficient to induce a significant decrease in the passive musculotendinous stiffness of the plantar flexor muscles. However, these authors reported that although stiffness decreased immediately after 2 min, 4 min, and 8 min of SS, the effects of stretching disappeared within 10 min. In turn, Mizuno et al. [44] showed that SS for 5 minutes at maximal dorsiflexion resulted in significantly increased range of motion that persisted for 30 min, but significant decreases in musculotendinous stiffness returned to baseline levels within 10 min. Taking into consideration the fact that the 3-km running was performed with an average time of 11 min, it can be suggested that the negative effects of SS on overall exercise performance was negligible. Thus, the negative effect of the SS in running performance might be restricted to the initial phase of a middle-distance event when the metabolic cues are less important for the running pacing strategy [42].

The current study does have some limitations. It is important to note that our SS treatment was composed of seven different exercises for the lower limbs, performed three times in a serial fashion at “high-intensity”, which was defined as scores of 8–9 on the Borg CR10 scale. This SS treatment may have resulted in a higher volume and intensity stretching protocol than those often used in the “real world”. In addition, our sample was composed by recreational runners, which have a lower endurance training volume and did not perform other training routines (i.e. strength or stretching training). In this manner, the effects of static stretching on the neuromuscular variables and pacing strategy in highly-trained subjects could be distinct from those observed in the present study. Thus, caution should be exercised in extrapolating these findings to runners with a higher training level.

In conclusion, the present study provides novel findings concerning the impact of stretching-induced impairment on neuromuscular function and pacing strategy. It was detected that SS resulted in a reduced capacity of the skeletal muscle to produce explosive force and a reduction in running speed during the acceleration phase of a time-trial. These results clearly show that, independent of the intention of the athletes to finish a race as quickly as possible, the neuromuscular system has a primary function in contributing to the chosen speed during the initial phase of the race, where the highest running speeds were reached.
